# Quantifying the impact of precipitation fluctuations on forest growth in Northeast China

**DOI:** 10.3389/fpls.2025.1570005

**Published:** 2025-05-20

**Authors:** Yue Hai, Tian Han, Yu Wang, Ruonan Li, Yanzheng Yang, Zhi Wen, Hua Zheng

**Affiliations:** ^1^ State Key Laboratory for Ecological Security of Regions and Cities, Research Center for Eco-Environmental Sciences, Chinese Academy of Sciences, Beijing, China; ^2^ University of Chinese Academy of Sciences, Beijing, China; ^3^ College of Geoscience and Surveying Engineering, China University of Mining and Technology-Beijing, Beijing, China; ^4^ Solux College of Architecture and Design, University of South China, Hengyang, Hunan, China

**Keywords:** Northeast China, semi-arid region, subhumid region, precipitation variability, forest growth

## Abstract

**Introduction:**

In the context of climate change, the escalating frequency of global precipitation fluctuations amplifies uncertainties in assessing the impact on forest ecosystem productivity. Hence, elucidating the dynamic relationship between precipitation characteristics and forest growth can provide effective management strategies for addressing climate change.

**Methods:**

This study utilizes precipitation data from 1982 to 2022 to construct the frequency and amplitude of precipitation fluctuations and analyzes the response of forest growth in northern China to these precipitation variations.

**Results:**

The growth of 13.7% of the region's forest is declining, with 8.1% of the area showing significant degradation. The core degradation zones for forest growth are located in semi-arid regions with precipitation frequencies ≥ 12 and amplitudes ≤ 60 mm and subhumid regions with precipitation frequencies ≥ 14 and amplitudes ≤ 65 mm. In the core semi-arid zone, deciduous broadleaf shrublands have greatest degraded area (2.8×104 ha), but deciduous needleleaf forests have the highest proportion of degradation (57.1%), while in the subhumid core degradation zone, deciduous broadleaf forests have the highest area (1.7×105 ha) and proportion of degradation (9.3%).

**Discussion:**

This study not only provides a novel perspective for evaluating forest ecosystem responses to precipitation characteristics, but also offers crucial theoretical support for advancing the implementation of Nature-based Solutions in practical applications.

## 1 Introduction

Forest growth converts solar energy into organic matter through photosynthesis, and the stronger the biomass accumulation capacity of healthy forest ecosystems (Liang and Wang, 2020). Precipitation is a decisive factor for tree growth and the ecological services of forest ecosystems. In semi-arid to subhumid regions, an annual rainfall of 400 mm represents a critical threshold for maintaining the health (Gessner et al., 2013; Chen et al., 2019; Liu, 2019), an increase in precipitation generally leads to a rise in forest growth (Hu et al., 2010; Reichstein et al., 2013; Li et al., 2024). However, changes in precipitation characteristics and the global hydrological cycle owing to climate change (Durack et al., 2012; IPCC, 2021; Ndehedehe et al., 2023) affect the dynamic response of forests to the original precipitation regimes. These changes impact not only forest growth but also the ecosystem services provided by forests, such as soil and water conservation, windbreaks, sand fixation, and climate regulation, especially in ecologically vulnerable areas. Therefore, clarifying the dynamic relationship between precipitation characteristics and forest growth is crucial for understanding the response of natural ecosystems to global climate change (Giorgi et al., 2019; Zhang et al., 2025).

Current research has analyzed the impact of precipitation on forest ecosystem from the perspectives of intensity and spatiotemporal scales (Guan et al., 2018; Wise and Dannenberg, 2022; Feng et al., 2024; Hu et al., 2025). The impact of rainfall intensity on forest significantly differs based on variations among stands. For example, as precipitation intensity increases, the productivity of temperate deciduous broadleaf forests may decrease, whereas that of deciduous needleleaf forests may increase (Fang et al., 2005). The temporal scale of precipitation primarily focuses on inter-annual and intra-annual variations (Viles and Goudie, 2003; Fatichi et al., 2012; Lyu et al., 2025). A greater inter-annual precipitation variability corresponds to lower vegetation growth (Liu et al., 2020a), and exceeding the variability threshold can lead to ecosystem mortality (Ye et al., 2013; Gherardi and Sala, 2015). Because of the phenological cycle of vegetation, seasonal precipitation characteristics are more significantly correlated with vegetation growth than annual totals (Robinson et al., 2013; Yuan et al., 2022; Chen and Zhang, 2023), and earlier rainfall during the growing season has a more pronounced effect on enhancing vegetation growth (Robinson et al., 2013; Lian et al., 2024; Zhu et al., 2025). Additionally, the increasingly extreme intra-annual distribution of precipitation significantly affects the response of forest ecosystems to inter-annual precipitation (Zhang et al., 2013; Zeppel et al., 2014). The spatial distribution of precipitation is mainly influenced by monsoons, but climate change has exacerbated its variability (Loo et al., 2015), making arid regions even drier and leading to the decline and even widespread mortality of forests in semi-arid regions (Liu et al., 2013).

Evidently, forest ecosystems exhibit complex dynamic responses to precipitation. However, current analyses of precipitation characteristics often focus on annual precipitation amounts or precipitation distribution within the year, with insufficient attention to the spectral properties and stochastic dynamics of precipitation variability. Hence, the impact of precipitation on forest has not been fully elucidated. Precipitation variability includes frequency and amplitude, referring to the number of precipitation events and the degree of variation in the amount of precipitation (Zhang et al., 2021), respectively. Precipitation frequency determines the interval at which forests receive precipitation; excessively frequent or infrequent precipitation events can trigger floods or droughts, suppress forest autotrophic respiration and photosynthesis, and affect forest growth (Craine et al., 2012; Marra et al., 2019). Precipitation amplitude directly affects the soil moisture supply; an appropriate amount of precipitation is beneficial for forest growth, whereas amounts exceeding or falling below certain thresholds can be detrimental (Zhang et al., 2018; Liu et al., 2024). Against the backdrop of climate change, precipitation events are expected to become more complex and vary in the future (IPCC, 2021), thereby increasing the risk to forest growth. Therefore, quantifying the impact of precipitation fluctuations on forest can enhance our understanding of the response and adaptive capacity of forest ecosystems to climate change and aid in the development of forest ecosystem management strategies.

This study introduces a novel approach for quantifying both precipitation frequency and amplitude as key indicators of precipitation fluctuation, and explores their impact on forest ecosystems dynamics. Specifically, the research aimed to analyze the dynamic characteristics of precipitation fluctuations and forest growth of forest ecosystems in Northeast China from 1982 to 2022. The objectives of the study were to: (1) establish indicators for assessing precipitation fluctuation and identify regions with precipitation variability and their characteristics, (2) elucidate the response relationship between precipitation fluctuation characteristics and forest dynamics and determine the thresholds at which precipitation fluctuation leads to significant differences in forest change trends, and (3) examine the growth trend conditions of different forest types within precipitation fluctuation threshold zones. The novelty of this study lies in the development of an integrated index for quantifying precipitation variability in terms of fluctuation frequency and amplitude. This study provides a scientific basis for addressing forest degradation under climate change, facilitating the implementation and application of Nature-based Solutions (NbS) in forest ecosystem management.

## 2 Study area and methods

### 2.1 Study area

**Figure 1:**
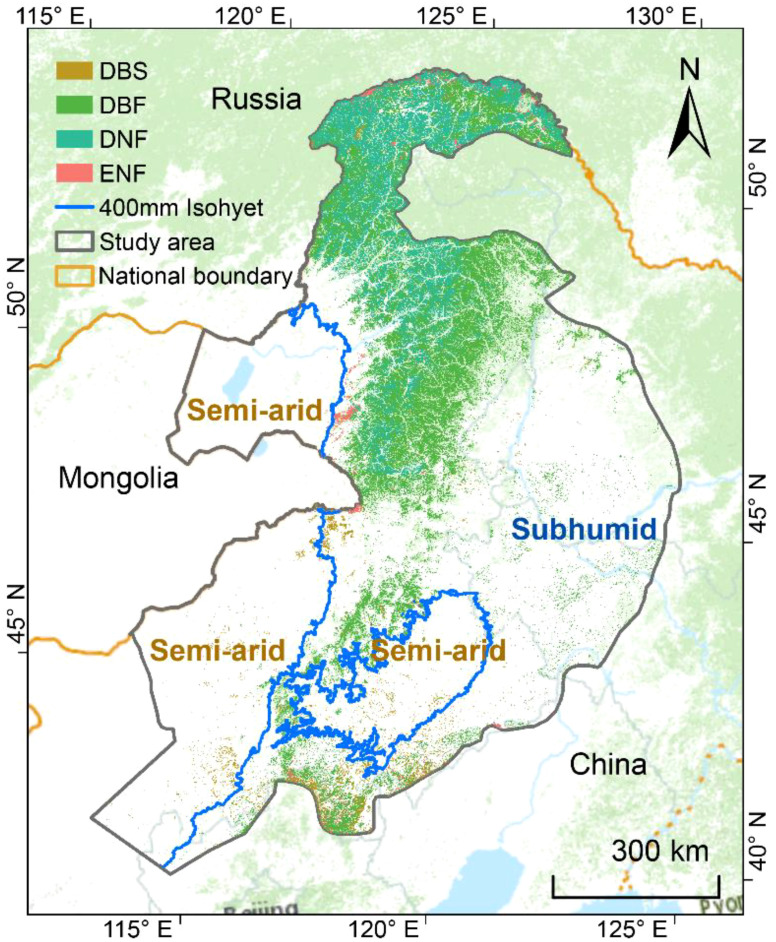
Spatial distribution of the main forest types and extent of the 400 mm isohyet averaged in the study area. DBS, Deciduous broadleaf shrubland, DBF, Deciduous broadleaf forest, DNF, Deciduous needleleaf forest, ENF, Evergreen needleleaf forest.

The northeast forest region accounts for 28.9% of the total forest area in China and is the largest natural forest area in the country (Nkonya et al., 2016). This region encompasses four main types of forests: deciduous broadleaf shrubland, deciduous needleleaf forest, evergreen needleleaf forest, and deciduous broadleaf forest. The forest ecosystems of northeast China, located in the semi-arid to humid transition zone, play a critical role in maintaining ecological security, and serve as a significant ecological barrier (Yu et al., 2011). Over the past few decades, the Northeast region has witnessed a substantial expansion in forest cover, driven by the implementation of large-scale ecological restoration programs, such as the Three-North Shelterbelt Forest Program and the Grain for Green Project (Nkonya et al., 2016; Song et al., 2022a). This expansion has been further facilitated by a shift in forest management policies, transitioning from timber production-oriented practices to ecological restoration and conservation (Yu et al., 2011). However, influenced by climate change, 400 mm isohyet (the forest-grassland boundary) has expanded eastward by 1850 km over the past few decades (Ma et al., 2016), profoundly affecting the ecosystem. To quantify the impact of precipitation fluctuations on the forest ecosystems of northeast China, we selected the area of spatial fluctuation around the 400mm isohyet as the study area, focusing on the forests within this region to analyze the dynamic relationship between precipitation characteristics and forest ecosystem. Based on multi-year precipitation data, we have divided the study area into semi-arid and subhumid zones (**Figure 1**).

### 2.2 Data sources

This study used unchanged stable forest ecosystems from 1980 to 2020 as the research subjects. The accuracy of land use data exceeds 95%, providing a comprehensive and objective understanding of the quantity and distribution of land use changes in China, as well as the patterns and characteristics of interactions among various land use types (Jiyuan et al., 2002). The NDVI data used in this study underwent a Bayesian calibration and correction procedure, ensuring the reliability of data quality (Pinzon and Tucker, 2014). Numerous studies have demonstrated a strong correlation between the normalized difference vegetation index (NDVI) and vegetation growth dynamics (Fensholt et al., 2012); hence, this study employed NDVI data to represent forest growth. To negate false NDVI signals due to winter snow cover, this study used values from the growing season (June to October) for analysis, with the sum of the NDVI values during the growing season indicating forest growth dynamics (Fang et al., 2005). Precipitation data were generated using the globally available 0.5°climate data released by CRU and the high-resolution global climate data provided by WorldClim, employing the delta spatial downscaling method. Validation was conducted using 496 independent meteorological observation points, which indicated a reduction in the mean absolute error of 25.7% (Peng et al., 2019). Annual precipitation data from 1982 to 2022 were used to extract 400 mm isohyet and calculate frequency and amplitude of precipitation fluctuations. Land use and NDVI data were sampled to a resolution of 1 km using precipitation grid data as the capture object. The specific descriptions of the data are presented in **Table 1**.

**Table 1:** Requisite data used in this study.

Data type	Acronym	Time	Spatial resolution	Temporal resolution	Data source
Precipitation	Pre	1982–2022	1 km	Mon	National Earth System Science Data Center, National Science & Technology Infrastructure of China (http://www.geodata.cn)
Land Use and Land Cover Change	LUCC	1980–2020	1 km	Year	Resource and Environmental Science Data Platform (https://www.resdc.cn/)
Normalized Difference Vegetation Index	NDVI	1982–2022	1/12°	15 d	National Oceanic and Atmospheric Administration, NOAA (https://ecocast.arc.nasa.gov/data/pub/gimms/)

### 2.3 Calculation and spatial distribution of precipitation fluctuation characteristics

Based on ecohydrological principles, the 400 mm isohyet serves as a critical ecological threshold for forest distribution (Rishmawi et al., 2016; Chen et al., 2019). To quantify precipitation fluctuations, we define the frequency of precipitation fluctuation as the number of times the 400 mm isohyet crosses a grid, reflecting the recurrence of drought events. Frequent precipitation fluctuations may intermittently limit soil water availability and impair root absorption of water, thereby exerting stress on forests (Gu et al., 2016; Ke, 2022). The amplitude of precipitation fluctuation indicates the deviation of annual precipitation relative to the 400 mm threshold, reflecting the intensity of drought. Greater amplitudes directly alter water availability and influence physiological processes such as photosynthesis and growth (Liu et al., 2020a; Yu et al., 2022). By analyzing these two indicators, this study reveals how the frequency and amplitude of precipitation fluctuations jointly regulate forest growth, providing a basis for predicting the responses of forest ecosystems to climate change.

**Figure 2:**
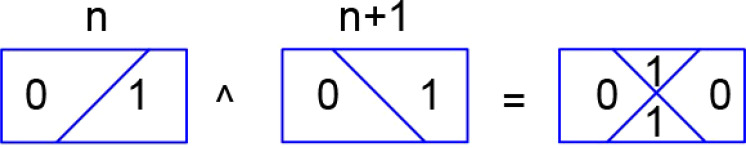
Calculation of precipitation fluctuation frequency in adjacent years.

Precipitation Fluctuation Frequency: Over a given time period, this represents the number of times the same isohyet line spatially overlaps in two consecutive years and is used to characterize the spatial variation of precipitation fluctuation (Quesada-Hernández et al., 2019). The calculation of precipitation fluctuation frequency was conducted as follows: First, based on annual precipitation raster data, the region was classified into two categories using 400 mm as a threshold: areas with annual precipitation ≥ 400 mm were assigned a value of 1, while other areas were assigned a value of 0. Next, a logical exclusive OR (XOR) operation was performed on the assigned data for adjacent years (**Figure 2**), while if they differed, the result was 1, thus reflecting changes in the spatial distribution of precipitation. Subsequently, the results of the XOR operation were summed across grids to obtain the precipitation fluctuation frequency for adjacent years. Finally, the precipitation fluctuation frequencies for all adjacent years within the study period were aggregated to derive the overall precipitation fluctuation frequency, which characterizes the spatial variability of precipitation during the entire study period. We define the study area as the region where the frequency of precipitation fluctuations is greater than 0, referred to as the precipitation fluctuation zone.

Precipitation Fluctuation Amplitude: Over a given period, the deviation relative to the 400 mm precipitation line was quantified. The formula used is as follows (**Equation 1**):


(1) $$D_{400}=\frac{\sum_{i=1982}^{n}(x_{i}-400)}{N}$$

where *x*_i_ represents the grid precipitation amount for year *i*, *n* represents the final year of observation, *N* represents the number of years observed and *D_400_* is the amplitude of the precipitation fluctuation. Areas with *D_400_* > 0 were classified as subhumid fluctuation zones, and areas with *D_400_* < 0 were classified as semi-arid fluctuation zones.

**Figure 3:**
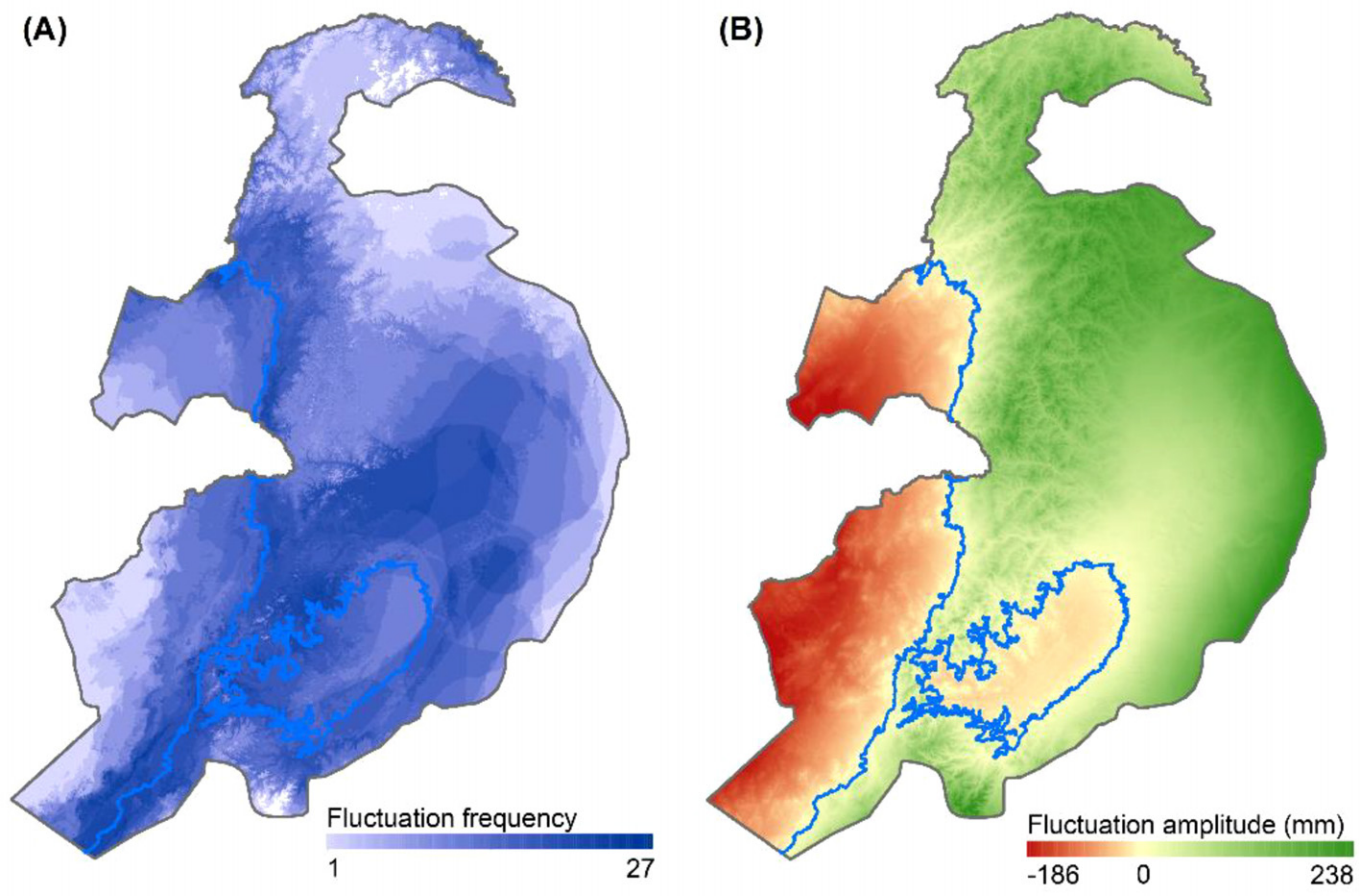
Spatial distribution of precipitation fluctuation **(A)** frequency and **(B)** amplitude.

**Figure 3** illustrates the spatial distribution of the frequency and amplitude of precipitation fluctuation within the study area from 1982 to 2022. The highest precipitation fluctuation frequency was 27, with 41.7% of the study area experiencing more than 14 fluctuations. The amplitude of precipitation fluctuation ranged from –186 to 238 mm; within this, the semi-arid fluctuation zone had a precipitation fluctuation amplitude ranging from –186 to 0 mm, while the subhumid fluctuation zone had an amplitude ranging from 0 to 238 mm.

### 2.4 Identification of forest degradation

In this study, the least squares method was used to analyze the trends in forest dynamics changes, a technique widely used in assessing vegetation dynamics (Lin et al., 2016; Mehmood et al., 2024). In assessing forest dynamics changes, this study employs residual analysis to filter out NDVI outliers, thereby effectively enhancing the accuracy and stability of the model estimates. The sign of the slope was employed to characterize the increase or decrease in forest, whereas the absolute value of the slope was used to represent the amplitude of the forest change. In addition, an F-test was used to assess the significance of the slope, and the classification levels were delineated (**Table 2**). The trends in forest changes were calculated as follows (**Equation 2**):

**Table 2:** Trend grade of forest dynamics.

Steady increase (SI)	Insignificant degradation (ID)	Significant degradation (SD)
*S_NDVI_* >0	*S_NDVI_* <0	*S_NDVI_* <0
Other	P>0.05	P ≤ 0.05


(2) $$S_{NDVI}=\frac{\text{n}\times \sum_{\text{i}=1}^{\text{n}}(i\times NDVI_{i})-\sum_{\text{i}=1}^{\text{n}}\text{i}\times \sum_{\text{i}=1}^{\text{n}}NDVI_{i}}{\text{n}\times \sum_{\text{i}=1}^{\text{n}}{\text{i}}^{2}-{\left(\sum_{\text{i}=1}^{\text{n}}\text{i}\right)}^{2}}$$

where *S_NDVI_* represents the slope of the linear regression equation for NDVI, *n* denotes the study period (y), and *NDVI_i_* represents the observed NDVI value for year *i*.

### 2.5 Change point detection

The Pettitt test is a rank-based non-parametric statistical test designed to detect change points within a data series (Pettitt, 1979; Hai et al., 2020). It has been used extensively to detect changes in hydrological and climatological records (Verstraeten et al., 2006; Zhang et al., 2009), and the method is as follows (**Equations 3–6**):


(3) $$\begin{array}{c} U_{t,\;\;n}=U_{t\;-1}+\sum_{j\;=\;1}^{n}sgn(x_{i}-x_{j}) \end{array}$$

(4) $$\begin{array}{c} sgn(x_{i}-x_{j})=\{\begin{array}{c} \begin{array}{cc} 1 & x_{i}-x_{j}\;>\;0 \end{array}\\ \begin{array}{cc} 0 & x_{i}-x_{j}\;=\;0 \end{array}\\ \begin{array}{cc} -1 & x_{i}-x_{j}\;<\;0 \end{array} \end{array} \end{array}$$

(5) $$\begin{array}{c} K=max|U_{t,\;\;n}|,t=1,2,\ldots ,n \end{array}$$

(6) $$\begin{array}{c} P≅2exp(-\;\frac{6K^{2}}{n^{3}\;+\;n^{2}}) \end{array}$$

where $U_{t,\;n}$ represents the defined statistic; *n* is the number of interval groups; $x_{i}$ and $x_{j}$ are the forest dynamics trends of the i-th and j-th groups, respectively; *K* is the year of the change point; if P≤ 0.05 , then the change point year is statistically significant. This study employs the Pettitt test to identify turning points in significantly degraded forest dynamics trends at precipitation fluctuation frequency and the amplitude. Additionally, a one-way analysis of variance (ANOVA) is conducted to ascertain the differences in forest dynamics trends before and after these turning points (P < 0.05), as referenced by Lix et al. (1996), where a lower mean of forest dynamics trends indicates more severe forest degradation. The article defines regions exhibiting greater forest degradation in relation to precipitation fluctuation frequency and amplitude as core areas, and evaluates the degradation trends of different forest types within these core areas.

## 3 Results

### 3.1 Forest degradation

**Figure 4:**
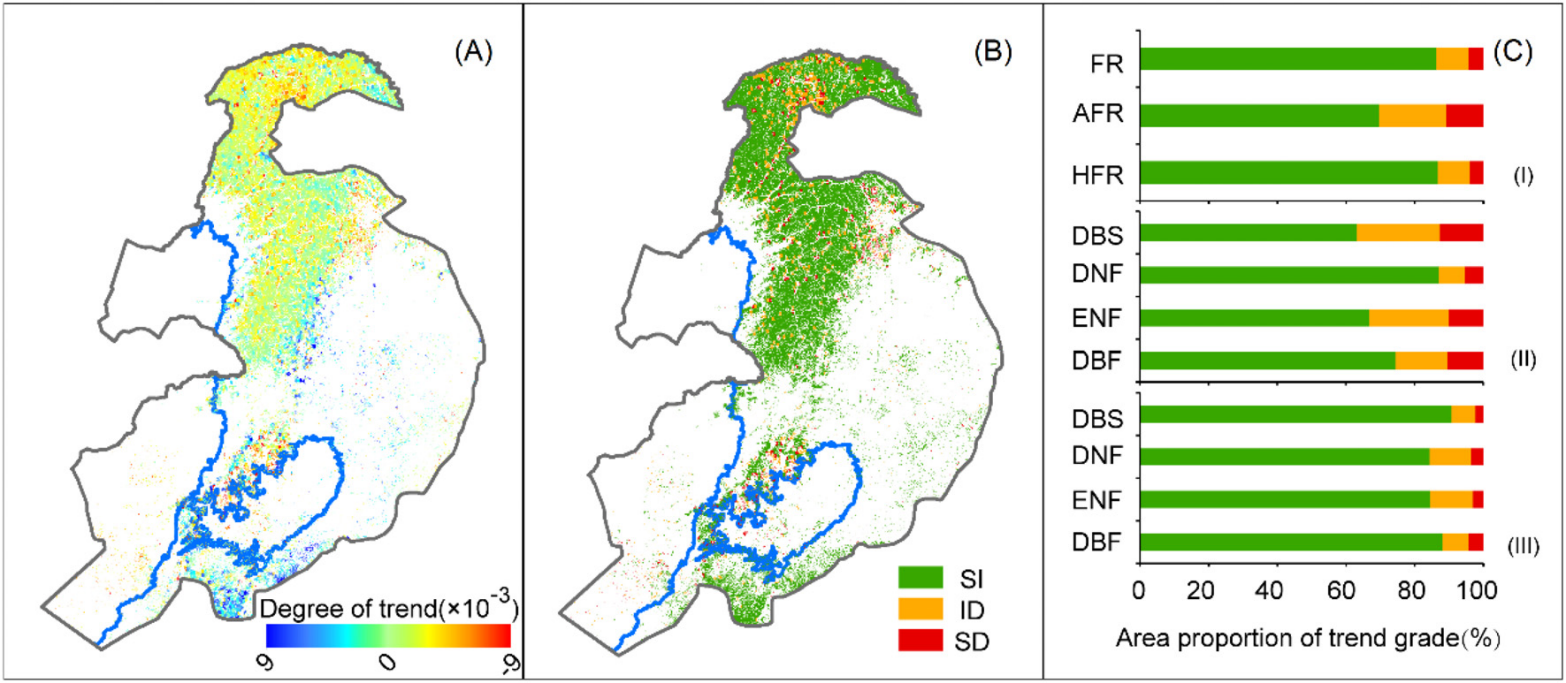
Spatial distribution of **(A)** forest dynamics trends and **(B)** trend significance and **(C)** trend-level statistics in terms of area and forest type in the (I) study area, (II) semi-arid fluctuation area, and (III) subhumid fluctuation area. FR, AFR, and HFR represent the entire study area, semi-arid, and subhumid fluctuation areas, respectively. SI indicates steady increase, ID indicates an insignificant decline, and SD indicates a significant decline. DBS, Deciduous broadleaf shrubland; DBF, Deciduous broadleaf forest; DNF, Deciduous needleleaf forest; ENF, Evergreen needleleaf forest.

In the precipitation fluctuation zone, the total area decreased by 2.5×10^6^ ha, accounting for 13.7% of the total forest area in these regions. Specifically, the forest degradation in the subhumid and semi-arid fluctuation zones was 2.3×10^6^ ha and 1.6×10^5^ ha, representing 13.2% and 30.4% of their respective forest areas. Further analysis revealed that 4.2% (7.5×10^5^ ha) of the forest area within the precipitation fluctuation zone experienced significant decline, primarily concentrated in the central part of the subhumid fluctuation zone and the transitional areas between the semi-arid and subhumid fluctuation zones. In detail, the significantly declined forest area accounted for 4.0% (7.0×10^5^ ha) and 10.9% (5.7×10^4^ ha) of the forest area in the subhumid and semi-arid fluctuation zones, respectively (**Figures 4A, B**). An analysis of the different forest types revealed that the semi-arid fluctuation zone has the largest proportion of significant decline in deciduous broadleaf shrublands (12.6%, 3.7×10^4^ ha), followed by deciduous broadleaf forests (10.3%, 1.4×10^4^ ha), evergreen needleleaf forests (9.9%, 2.6×10^3^ ha), and deciduous needleleaf forests (5.3%, 3.8×10^3^ ha). In the subhumid fluctuation zone, deciduous broadleaf forests show the highest proportion of significant decline at 4.3% (4.6×10^5^ ha), followed by deciduous needleleaf forests (3.7%, 2.1×10^5^ ha), evergreen needleleaf forests (3.1%, 1.3×10^4^ ha), and deciduous broadleaf shrublands (2.3%, 1.5×10^4^ ha) (**Figure 4C**).

### 3.2 Qualitative impact of precipitation change frequency and amplitude on forest dynamics

**Figure 5:**
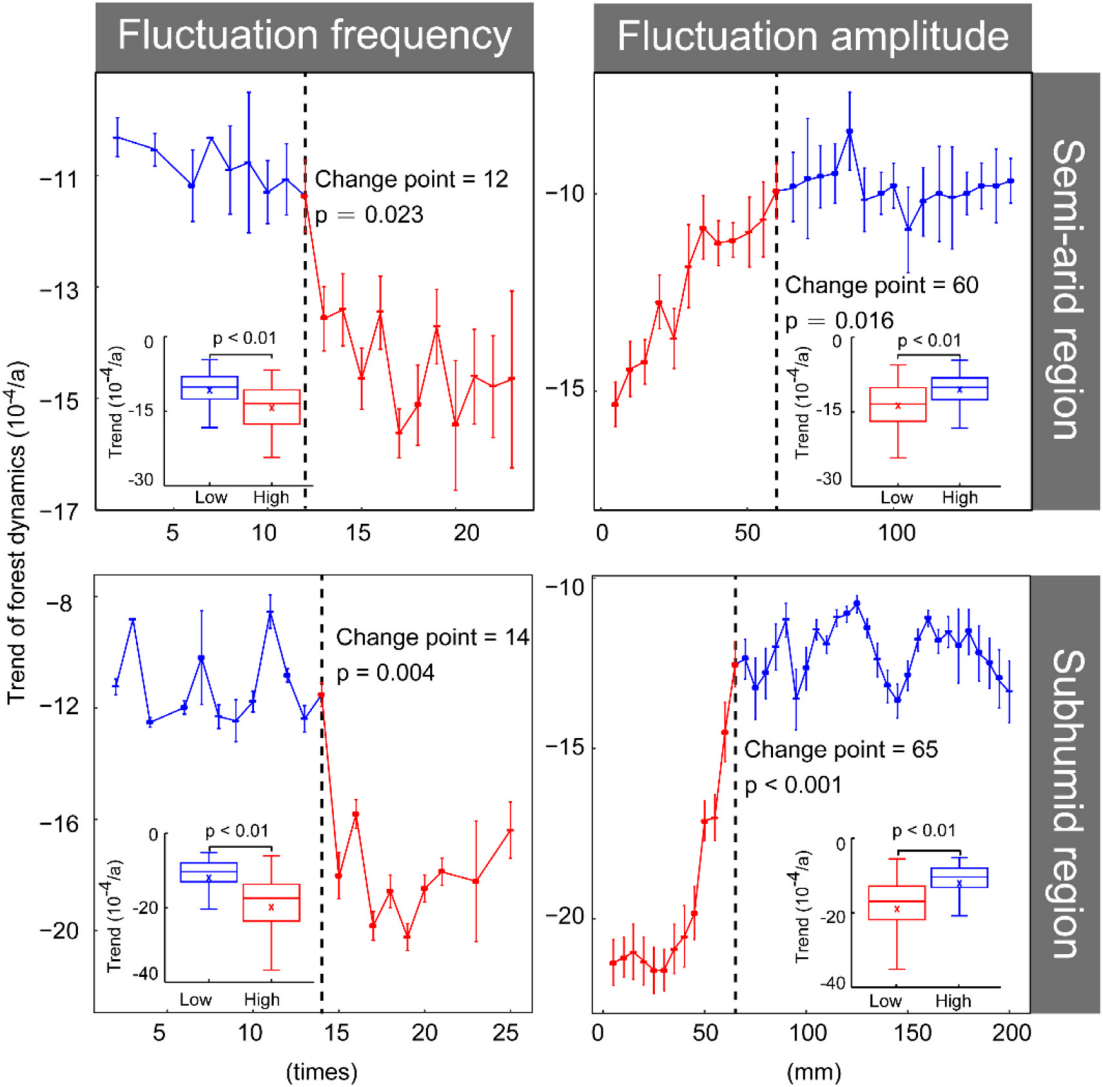
Forest dynamics trend of significantly degraded forests based on the frequency and amplitude of precipitation fluctuation. The dashed line indicates the threshold for abrupt changes, the red dotted line represents the significant relationship between forest trends and changes in precipitation frequency and amplitude, while the blue dotted line represents the absence of a significant relationship between forest trends and changes in precipitation frequency and amplitude. The subgraph shows the differences in the trend of forest growth before and after the threshold for abrupt changes.

Overall, forest dynamics exhibited a stability trend followed by a decline as the precipitation fluctuation frequency increased. Meanwhile, an initial rise followed by stabilization as the precipitation fluctuation amplitude increased (**Figure 5**). This indicates that areas with high-frequency and low-amplitude precipitation fluctuations have lower forest growth and more dramatic change, whereas areas with low-frequency and high-amplitude precipitation fluctuations have higher forest growth and are relatively stable. For precipitation fluctuation frequency, the inflection points for forest growth did not significantly differ across different climatic zones, being 12 and 14 times in semi-arid and subhumid areas, respectively. Furthermore, the relationship between forest dynamics and precipitation fluctuation amplitude had an inflection point in semi-arid zones that was lower than that in subhumid zones (60 and 65 mm, respectively) (**Figure 5**).

### 3.3 Differences in forest dynamics in the core areas

**Figure 6:**
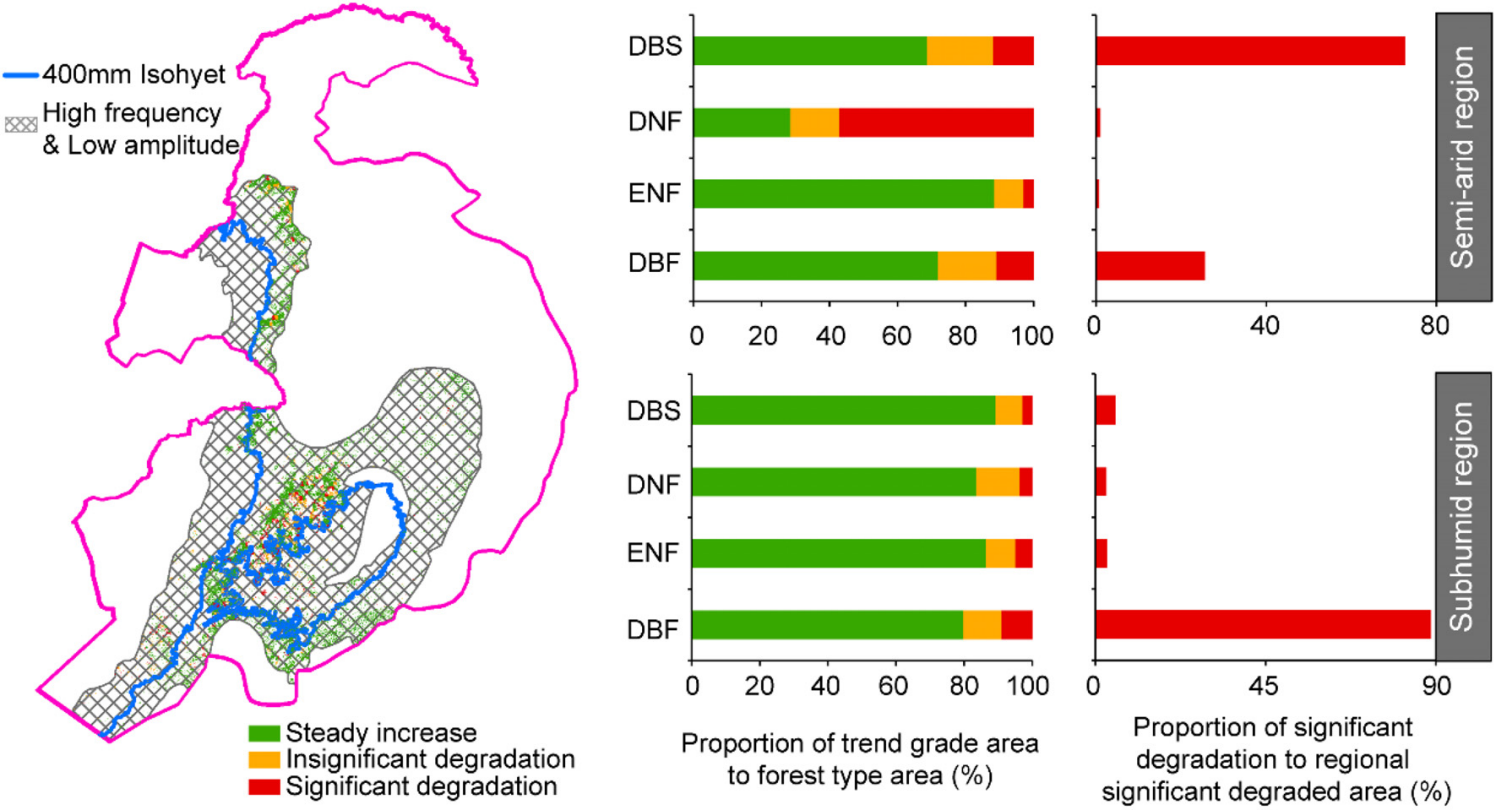
Spatial distribution of forest dynamics trend in areas with high-frequency and low-amplitude precipitation fluctuation. The histogram shows the trend-level statistics of different forest types in the two fluctuation zones. DBS, Deciduous broadleaf shrubland; DBF, Deciduous broadleaf forest; DNF, Deciduous needleleaf forest; ENF, Evergreen needleleaf forest.

This study further identified the relationships between precipitation characteristics and forest types in the core areas characterized by high-frequency but low-amplitude precipitation fluctuations (**Figure 6**). In the semi-arid fluctuation zone, the most significant decline in forest dynamics was observed in deciduous needleleaf forests (57.1%), followed by deciduous broadleaf shrublands (12.0%), deciduous broadleaf forests (11.0%), and evergreen needleleaf forests (2.9%). In terms of area, deciduous broadleaf shrublands showed the largest significant decline, accounting for 72.6% of the total significantly degraded area, followed by deciduous broadleaf forests (25.6%), deciduous needleleaf forests (1.0%), and evergreen needleleaf forests (0.8%). In the sub-humid fluctuation zone, deciduous broadleaf forests exhibited the largest proportion of significant decline (9.1%), followed by evergreen needleleaf forests (4.9%), deciduous needleleaf forests (3.6%), and deciduous broadleaf shrublands (2.8%). In terms of area, deciduous broadleaf forests accounted for the largest significant decline, comprising 88.7% of the degraded area, followed by deciduous broadleaf shrublands (5.2%), evergreen needleleaf forests (3.2%), and deciduous needleleaf forests (2.9%).

## 4 Discussion

### 4.1 Identified forest degradation

This study analyzed forest dynamics trends in the precipitation fluctuation zone of Northeastern China, revealing that 13.7% of the forest area experienced a decline, with 4.2% showing significant downward trends, and notable differences were observed between semi-arid and subhumid regions (**Figure 4**). Forest degradation in Northeast China has been corroborated by multiple studies. For instance, long-term (1982–2012) and segmented (1982–1997 and 1997–2012) analyses based on GIMMS NDVI data indicates that 38.91–46.21% of vegetation in China has exhibited a declining trend, with significant decreases in the Northeast region (Liu et al., 2015). Furthermore, dendrochronology analyses indicate that since 1994, forest growth trends in China’s semi-arid to subhumid forests have generally decreased (Liu et al., 2013), with the correlation between forest growth and precipitation being significantly stronger than that with temperature (Poulter et al., 2013). This phenomenon may be closely related to the reductions in precipitation and the intensification of droughts caused by climate change. Studies have shown that both the frequency and intensity of precipitation decrease and drought events in Northeastern China are on the rise (Sheffield et al., 2009; Huang et al., 2016), and more frequent and severe droughts significantly increase the likelihood of reduced forest growth (Allen et al., 2010). Notably, forests in ecologically vulnerable areas exhibit high sensitivity to climate change (Liu and Yin, 2013; Shi et al., 2021). However, there are differences in the adaptive capacities of different vegetation zones to climate change (Maherali et al., 2004; Jiang et al., 2023), which may be an important reason for the differences observed in forest dynamics trends between semi-arid and subhumid regions.

In terms of forest community structure, evergreen needleleaf forest exhibit the highest rates of degradation in semi-arid regions, which may be related to their higher water requirements (Wang et al., 2018). Under drought conditions, their resilience is relatively weak (Lv et al., 2022), making them more susceptible to water stress. Deciduous needleleaf forests exhibit comparable degradation rates in semi-arid and subhumid regions, demonstrating robust adaptive capacity. This resilience may be attributed to the fact that, under drought conditions, deciduousness reduces water transpiration (Zeng et al., 2025), thereby enhancing their drought resistance. Deciduous broadleaf forests exhibit significant degradation in arid environments, primarily due to their high sensitivity to drought stress (Kang et al., 2025). Reduced moisture availability leads to premature leaf abscission (Singh and Kushwaha, 2016), thereby restricting the normal growth and development of trees and exacerbating the process of forest degradation. Deciduous broadleaf shrublands in semi-arid regions are more prone to degradation than those in subhumid areas. This heightened degradation is attributed to the shrubs’ lack of effective self-regulation mechanisms under more adverse conditions (Kooch et al., 2022).

### 4.2 Role of precipitation characteristics on forest growth and change point

This study identifies significant change points in forest dynamics that correspond to the frequency and amplitude of precipitation fluctuations. Specifically, regions characterized by high-frequency and low-amplitude precipitation fluctuations experience more severe forest degradation than others, due to the resulting instability of available water resources for vegetation. In subhumid regions, the high frequency of precipitation fluctuations indicates that the precipitation amount falls below the critical threshold of 400 mm in multiple years. These frequent drought conditions weaken the moisture supply in forest soils (Liu et al., 2020a), making it difficult for trees to acquire sufficient water to maintain normal physiological activities, thereby exacerbating forest degradation. In semi-arid regions, areas characterized by large precipitation fluctuation amplitudes are primarily dominated by herbaceous plants, shrubs, or sparse forests rather than forest ecosystems. These types of vegetation exhibit strong adaptability and resilience to precipitation fluctuations (Zhao et al., 2015; Hu et al., 2021), which results in a relatively minor impact of such fluctuations on the overall ecosystem.

In addition to the amount of precipitation (e.g., surplus or deficit), the temporal characteristics of precipitation (e.g. extreme events, consecutive droughts, and precipitation variability) also play a significant role in ecosystem dynamics (Liu et al., 2020a). The impacts of precipitation characteristics on forest dynamics exhibit regional differences and vary among forest types (Knapp et al., 2002; Heisler-White et al., 2008; Guan et al., 2018). The direction of the impact of extreme precipitation events on forest growth also varies by geographical region. For example, in arid regions, extremely wet conditions can stimulate forest growth to compensate for the decline caused by extreme drought (Jiang et al., 2019). In humid regions, extreme precipitation can negatively affect forest growth and even lead to tree mortality (Zhao et al., 2005). Moreover, the degree of impact of precipitation surplus or deficit on forest dynamics varies regionally. For example, in arid regions, forest growth generally increases in wet years, while the decrease in dry years is relatively small; in humid regions, the increase in wet years is smaller, while the decrease in dry years is relatively larger (Al-Yaari et al., 2020). In this study, the core areas of declining forest growth were primarily located in areas with high-frequency and low-amplitude precipitation fluctuation distributed across both the semi-arid and subhumid zones (**Figure 5**). The diversity in the impact of precipitation characteristics on forest types is reflected in the dynamic relationship between drought persistence and forest growth. For instance, consecutive droughts have exacerbated the decline in needleleaf forests, whereas the impact of initial droughts is more pronounced in broadleaf forests (Anderegg et al., 2020; Liu et al., 2020b). Deciduous broadleaf shrublands had a lower proportion of significant decline in the core areas compared with that in deciduous broadleaf, evergreen needleleaf, and deciduous needleleaf forests (**Figure 6**). The above analysis indicated that quantifying precipitation characteristics from different perspectives can better identify the dynamic response of forests to precipitation.

### 4.3 Implications for forest management

By exploring the impact of precipitation fluctuation characteristics on forest growth, this study quantified the variability in the growth response of different forest types to precipitation fluctuations. These findings provide critical insights for shaping effective forest conservation and restoration policies, particularly in the context of climate variability and change.

Region-specific management strategies should be developed to address unique environmental conditions. For instance, in forest-steppe ecotones, ecological afforestation projects should prioritize drought-resistant species and optimize shrub density to enhance water-use efficiency (Chen et al., 2015), thereby addressing current and future climate changes (Deng et al., 2019; Xiao et al., 2021). Forest composition should be diversified by selecting resilient forest types. Deciduous broadleaf forests, which cover the largest areas and higher degradation rate, often shed leaves to cope with drought, reducing water loss but resulting in relatively poor drought resistance (Lv et al., 2022). Therefore, selecting drought-resistant deciduous broadleaf species is essential to respond to precipitation changes. Because of their lower evapotranspiration rates, evergreen needleleaf forests show strong resistance in areas of fluctuating precipitation (Song et al., 2022b), suggesting that increasing their proportion can mitigate growth decline and enhance the stability of future forest ecosystems. Afforestation practices must consider the water adaptability of trees, and projects should incorporate water-adaptive measures into planning and implementation. Widespread forest growth decline (**Figure 4**) has been observed, with related studies showing that the afforestation survival rate in China’s Three-North Shelterbelt Program is only 60% (Xiao et al., 2021). The failure to account for water adaptability in afforestation is a significant factor contributing to large-scale plantation losses (Deng et al., 2019).

In the future, global warming will intensify hydrological cycle (IPCC, 2021; Ndehedehe et al., 2023), and extreme weather will alter precipitation characteristics (Jiang et al., 2019), inevitably having a profound impact on global forest and forest ecosystem services and functions (Aleixo et al., 2019). In the face of complex environmental challenges, continuous monitoring of forest dynamics and precipitation patterns is essential to inform adaptive management strategies. Policymakers should invest in research to better understand the interactions between climate change, precipitation fluctuations, and forest growth. In particular, forests in northern China are mostly even-aged monoculture plantations, which have poorer ecosystem stability and resilience compared to natural forests (Huang et al., 2016), and will face significant risks in adapting to future climate change (**Figure 6**). In the management of ecological projects and policy-making processes in northern China, increasing forest tree species diversity can be a valuable strategy to enhance the resilience of forests against frequent and intense precipitation changes, and this positive effect is most pronounced in arid zone forests (Peng et al., 2020; Liu et al., 2022).

### 4.4 Limitations and future research directions

Despite the widespread use of NDVI as an indicator for monitoring forest growth dynamics, its effectiveness varies significantly across different ecological systems. In humid regions with high vegetation cover, NDVI tends to saturate, rendering it incapable of detecting subtle changes in plant growth. Conversely, in arid areas with low vegetation cover, soil background signals can obscure vegetation signals, leading to insufficient sensitivity of NDVI for accurately detecting vegetation changes. In the semi-arid and semi-humid transitional zones addressed in this study, where vegetation density is moderate, NDVI proves effective in capturing variations in plant growth. Clearly, selecting appropriate remote sensing indices based on the specific climatic and vegetation characteristics is crucial for accurately representing forest growth conditions. It is recommended to use the Enhanced Vegetation Index (EVI) in areas with high vegetation cover and the Modified Soil-Adjusted Vegetation Index (MSAVI) in arid regions to overcome the inherent limitations of NDVI in extreme biomes.

This study focuses on forest areas that remained unchanged between 1980 and 2020. While this approach effectively controls for the natural processes of forest succession influencing growth, it may underestimate the true impact of precipitation fluctuations on forest growth. By concentrating solely on stable forest regions, the research overlooks the reductions in forest area caused by degradation, logging, or land cover change during this period, potentially limiting the findings’ capacity to fully capture the dynamic changes within forest ecosystems. Future studies should integrate considerations of both increases and decreases in forest area to formulate more comprehensive forest management and conservation strategies.

## 5 Conclusion

The results of this study clearly indicated that the frequency and amplitude of precipitation fluctuations were significant determinants of the state of forest ecosystems, with high-frequency and low-amplitude precipitation fluctuations being particularly impactful, as 8.14% of forests exhibited notable degradation. This had to be taken into consideration in the context of increasingly complex and variable precipitation characteristics in the past. With the use of NDVI, precipitation, and land use data, our findings indicated that in the semi-arid core degradation zone, deciduous broadleaf shrublands covered the largest degraded area, whereas deciduous needleleaf forests had the highest proportion of degradation; In the subhumid core degradation zone, deciduous broadleaf forests had the highest area and proportion of degradation, amounting to 1733 km² and 9.13%, respectively. This study evaluated the response of forest ecosystems to precipitation characteristics in terms of fluctuation frequency and amplitude, providing a basis for the restoration and management of forest ecosystems in arid regions, and for addressing climate change.

## Data Availability

The original contributions presented in the study are included in the article/supplementary material. Further inquiries can be directed to the corresponding author.
